# An Overview of Systematic Reviews of Using Chinese Medicine to Treat Polycystic Ovary Syndrome

**DOI:** 10.1155/2021/9935536

**Published:** 2021-05-28

**Authors:** Linjing Wang, Runyu Liang, Qiang Tang, Luwen Zhu

**Affiliations:** ^1^Affiliated No. 2 Hospital, Heilongjiang University of Chinese Medicine, No. 411 Guogeli Road, Nangang District, Harbin, Heilongjiang 150001, China; ^2^Heilongjiang University of Chinese Medicine, Harbin, Heilongjiang 150040, China

## Abstract

**Objective:**

This review sought to evaluate the strength and validity of the existing evidence for the use of Chinese medicine for the treatment of polycystic ovary syndrome (PCOS).

**Methods:**

We retrieved systematic evaluations and meta-analyses of randomized controlled trials (RCTs) evaluating Chinese herbal interventions in polycystic ovaries, including the use of decoctions or Chinese patent medicines. The quality of these systematic evaluations was assessed using AMSTAR2 tools, and ovulation rate, pregnancy rate, effective rate, serum hormones (testosterone, luteinizing hormone, and follicle-stimulating hormone), and adverse reactions were recorded. Finally, the reliability of each result was evaluated according to the GRADE system. *Data Sources*. PubMed, Embase, Cochrane Library, China National Knowledge Infrastructure (CNKI), Wanfang Data, CQVIP, and SINOMED databases were searched up to January 1, 2021. *Outcomes.* A total of 18 publications were included, all of which showed that PCOS symptoms were improved with Chinese medicine compared with control groups. However, most of the evaluations did not have good research designs and had issues with the analysis of their results. The reliability of most outcome measures was rated low or very low, and it is presumed that the reliability of the results was low due to the poor quality of the RCTs.

**Conclusions:**

At present, there is insufficient evidence to suggest that improved efficacy is achieved by the combined use of Chinese and Western medicine compared with Western medicine alone in treating PCOS. Therefore, it is recommended that multicenter, large-sample RCTs adopting standard designs and rigorous methods be carried out in the future while introducing standardized assessment plans for the systematic review of clinical trials so as to improve the quality of the resulting clinical evidence.

## 1. Introduction

Polycystic ovary syndrome (PCOS) is a common endocrine and metabolic disorder among women of reproductive age, and it is the main cause of anovulatory infertility [1]. Its major manifestations include ovulation disorders, irregular menstrual cycles, high levels of androgens, and depression and other emotional disorders [2, 3]. Meanwhile, patients often develop insulin resistance, obesity, and other metabolic disorders, which puts women with PCOS at a very high risk of developing diabetes [4, 5]. In addition, PCOS patients are prone to serious complications, and their risk of cardiovascular disease is higher than the general population [6], and they tend to suffer from fatty liver, metabolic syndrome, and other diseases [7, 8]. Clinically, PCOS is often managed by controlled ovulation stimulation and androgen suppression. However, while these methods can achieve certain therapeutic effects, they may also produce adverse effects such as vomiting and diarrhea [9]. Therefore, some Chinese herbal medicines are popular because of their low levels of side effects and adverse reactions even after long-term use [10]. These include cinnamon and other medicines that appear to work at the level of sex hormones and that are believed to play a role in the regulation of the menstrual cycle [11, 12]. In addition, Chinese medicine can increase the ovulation rate and improve hormone levels by regulating qi, blood, yin, and yang in all phases of the menstrual cycle [13]. Although several recent studies have systematically reviewed the efficacy of Chinese medicine for the treatment of PCOS, the quality of their methods and conclusions requires further verification. In this study, the methodological quality and the quality of evidence of existing systematic reviews on Chinese medicine for the treatment of PCOS were evaluated using the AMSTAR2 and GRADE systems in order to provide guidance for planning future studies on this subject.

## 2. Materials and Methods

### 2.1. Protocol and Registration

This study provides an overview of systematic evaluations based on existing recommendations and in accordance with the guidelines of the Preferred Reporting Items for Systematic Reviews and Meta-Analyses (PRISMA) [14]. The review was registered in the PROSPERO database (CRD42021242641).

### 2.2. Inclusion and Exclusion Criteria

#### 2.2.1. Research Types

This study included systematic reviews and meta-analyses of randomized controlled trials (RCTs) written in Chinese or English.

#### 2.2.2. Research Subjects

Articles that enrolled patients with confirmed diagnoses of PCOS were included in this study, and there was no restriction on the age of the patients or the course of the disease.

#### 2.2.3. Interventions

Articles included in this study must have adopted an intervention in which the experimental group was treated with either Chinese medicine alone or in combination with Western medicine, while the control group was treated with Western medicine alone or placebo. There were no restrictions on the type of Chinese or Western medicine used.

#### 2.2.4. Outcome Indicators

This study included articles with the primary indicators of live birth rate, pregnancy rate, ovulation rate, and clinical efficiency and the secondary indicators of adverse effects and serum hormone levels (testosterone (T), luteinizing hormone (LH), follicle-stimulating hormone (FSH), and LH/FSH).

#### 2.2.5. Exclusion Criteria

Exclusion criteria were as follows: (i) articles that were not systematic reviews or meta-analyses, (ii) meta-analyses of protocols and network meta-analyses, (iii) duplicate publications, (iv) trials containing other treatments such as acupuncture, (v) studies with outcome indicators that did not include at least two of the primary indicators listed above, (vi) studies with an inappropriate search strategy or coverage of fewer than two databases, and (vii) studies with erroneous conclusions or data.

### 2.3. Search Strategy

Systematic reviews and meta-analyses were searched for in the PubMed, Cochrane Library, Embase, CNKI, Wanfang Data, QVIP, and SINOMED databases. The search period was set from the inception of the database to January 1, 2021, with literature only in Chinese or English included. Two examples of the search strategies are shown in Table 1. A manual search of protocol registries and other unpublished sources was also performed as a supplement to avoid missing relevant literature.

### 2.4. Literature Screening and Data Extraction

Two researchers conducted independent screening of the literature by merging the search results and then removing duplicates using Endnote X9 (Clarivate Analytics, USA), followed by literature screening based on the aforementioned criteria. Once the cross-checks were completed, the two researchers performed the data extraction and quality evaluation separately. Disagreements were resolved after discussion between the two researchers, with the assistance of a third researcher if required.

### 2.5. Evaluation Methods

#### 2.5.1. Evaluation of the Methodological Quality

The methodological quality of the included studies was evaluated using the AMSTAR2 tool [15]. The quality of the 16 items in the tool was rated individually for each study, with items 2, 4, 7, 9, 11, 13, and 15 being prioritized.

#### 2.5.2. Evaluation of the Quality of the Evidence

The quality of the evidence of the included studies was evaluated using the GRADE evaluation system [16]. The limitation, inconsistency, indirectness, imprecision, and publication bias of each outcome indicator of the systematic reviews were objectively evaluated and assigned a confidence rating.

## 3. Results

### 3.1. Literature Screening Process and Results

**Figure 1:**
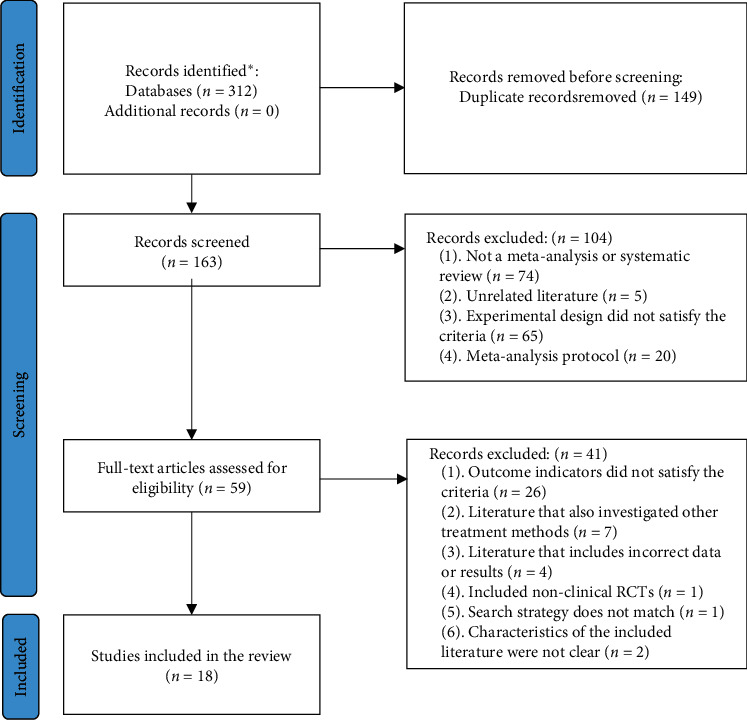
Literature screening process. ^∗^PubMed (*n* = 33), Embase (*n* = 15), Cochrane Library (*n* = 1), CNKI (*n* = 70), Wanfang (*n* = 79), QVIP (*n* = 58), and SINOMED (*n* = 56).

The initial search yielded 312 relevant articles, and after removing 149 duplicates, a total of 163 articles were screened, including 122 Chinese and 35 English articles. After initial screening and rescreening, 18 articles were eventually included in this study; all of which were written in Chinese. The detailed literature screening process is shown in Figure 1 [14].

### 3.2. Characteristics of the Included Studies

Of the 18 systematic reviews/meta-analysis included in this review [17–34], 14 were journal articles [17, 19–24, 27, 28, 30–34], and four were theses [18, 25, 26, 29]. None of the studies were registered in the Cochrane Library, PROSPERO, or the like. The basic characteristics of the included studies are listed in Table 2.

### 3.3. Quality Evaluation of the Included Systematic Reviews

#### 3.3.1. Evaluation of Methodological Quality

The AMSTAR2 assessment showed that five items (1, 4, 5, 8, and 9) were relatively complete and were reported by ≥70% of the articles, while there were five items (2, 3, 7, 10, and 16) that were not reported by any article (0%). The percentages of articles reporting the prioritized items were as follows: Item 2: 0%, Item 4: 100%, Item 7: 0%, Item 9: 89%, Item 11: 39%, Item 13: 56%, and Item 15: 67%. The overall credibility of the included systematic reviews was very low. Specific evaluation results are listed in Table 3.

#### 3.3.2. Evaluation of the Quality of the Evidence

The quality-of-evidence ratings for the outcome indicators of the included reviews were moderate, low, or very low quality. All quality-of-evidence ratings were downgraded due to research limitations because the methods adopted by these reviews to include RCTs were significantly biased and featured irregular, incorrect, or even semirandomized methods. Moreover, most RCTs did not state the use of a blinded method. In terms of serum hormone levels, significant heterogeneity was observed in the levels of T, LH, FSH, and LH/FSH, which contributed substantially to the inconsistencies in the results. Furthermore, imprecise conclusions and publication bias arising from the wide 95% confidence intervals and the small number of RCTs in some reviews had a negative impact on the quality of the evidence. Specific GRADE quality-of-evidence ratings are listed in Tables 4–11.

### 3.4. Primary Outcome Indicators

#### 3.4.1. Live Birth Rate

None of the included systematic reviews listed live birth rate as an outcome indicator.

#### 3.4.2. Pregnancy Rate

A total of 17 systematic reviews reported on the pregnancy rate. Of these, 10 compared the pregnancy rate after combined treatment with Chinese and Western medicine with that achieved after treatment with Western medicine alone [18, 20, 23, 24, 28, 30–34]; two compared the pregnancy rate achieved after treatment with Chinese medicine alone with that after treatment with Western medicine alone [25, 29], and the remaining five compared the pregnancy rate achieved after combined treatment with Chinese and Western medicine with that after treatment with either Chinese or Western medicine alone [17, 19, 21, 22, 26]. All of the results suggested a higher pregnancy rate in the experimental group than in the control group. In addition, only the subgroup analysis of one review [22] indicated that the combined value of the experimental group after treatment with Chinese medicine alone on the pregnancy rate crossed the line of null effect when compared with that of Western medicine.

#### 3.4.3. Ovulation Rate

A total of 13 systematic reviews reported on the ovulation rate. Of these, nine reviews compared the ovulation rate after combined treatment with Chinese and Western medicine with that of Western medicine alone [18, 23, 24, 27, 28, 30–33]. Of these, only one suggested that the combined value of the experimental group crossed the line of null effect [18], whereas the rest showed a higher ovulation rate in the experimental group. Conversely, one review compared the ovulation rate after treatment with Chinese medicine alone with that of Western medicine alone and showed that not only did the combined value of Chinese medicine alone cross the line of null effect when compared with Western medicine but the center of the diamond also favored the control group [25]. The remaining three studies compared the ovulation rate after the combined treatment with Chinese and Western medicine with that of either Chinese or Western medicine alone [17, 19, 26]. Of these, the subgroup analysis of one article showed no statistical significance between the ovulation rates after treatment with Chinese and Western medicine [26]. Therefore, the existing literature does not support the hypothesis that Chinese medicine is more effective than Western medicine in improving the ovulation rate of patients with PCOS.

#### 3.4.4. Clinical Efficiency

A total of 15 systematic reviews reported on clinical efficiency. Of these, nine compared the clinical efficiency of the combined use of Chinese and Western medicine with that of Western medicine alone [18, 20, 23, 27, 28, 32–34]; one compared the clinical efficiency of Chinese medicine alone with that of Western medicine alone [29], and the remaining five compared the clinical efficiency of the combined use of Chinese and Western medicine with that of either Chinese or Western medicine alone [17, 19, 21, 22, 26]. Although all results showed that the efficiency in the test group was higher than that in the control group, two articles indicated that the combined value of Chinese medicine alone crossed the line of null effect when compared with Western medicine [21, 22].

#### 3.4.5. Testosterone Level

A total of 10 systematic reviews reported on the T level. Of these, 7 compared the T level after the combined treatment with Chinese and Western medicine with that of Western medicine alone [18, 27, 28, 30–33]; 2 compared the T level after treatment with Chinese medicine alone with that of Western medicine alone [25, 29]; and 1 compared the T level after combined treatment with Chinese and Western medicine with that of either Chinese or Western medicine alone [26]. Except for 1 article [26], all studies suggested that the T level of the test group was significantly lower than that of the control group.

#### 3.4.6. Luteinizing Hormone Level

A total of 10 systematic reviews reported on the LH level. Of these, 7 compared the LH level after the combined treatment with Chinese and Western medicine with that of Western medicine alone [18, 20, 27, 28, 30, 32, 33]; 2 compared the LH level after treatment with Chinese medicine alone with that of Western medicine alone [25, 29], and 1 compared the LH level after the combined treatment with Chinese and Western medicine with that of either Chinese or Western medicine alone [26]. All results indicated that the LH level of the experimental group was lower than that of the control group.

#### 3.4.7. Follicle-Stimulating Hormone Level

A total of six systematic reviews reported on the FSH level. Of these, four compared the FSH level after the combined treatment with Chinese and Western medicine with that of Western medicine alone [27, 28, 30, 33]. All but one study indicated a lower FSH level in the test group than in the control group [33]. In contrast, one study compared the FSH level after treatment with Chinese medicine alone with that of Western medicine alone and showed no significant differences [29]. Another study [26] compared the FSH level after combined treatment with Chinese and Western medicine with that of either Chinese or Western medicine alone and also showed no significant differences.

#### 3.4.8. LH/FSH Level

A total of six systematic reviews reported on the LH/FSH level. Of these, four compared the LH/FSH level after the combined treatment with Chinese and Western medicine with that of Western medicine alone [18, 20, 27, 32]; one compared the LH/FSH level after treatment with Chinese medicine alone with that of Western medicine alone [29]; and one compared the LH/FSH level after the combined treatment with Chinese and Western medicine with that of either Chinese or Western medicine alone [26]. All results suggested that the LH/FSH level was lower in the experimental group than in the control group.

#### 3.4.9. Adverse Reactions

A total of seven systematic reviews reported adverse reactions. Of these, two compared the adverse effects of the combined use of Chinese and Western medicine with the use of Western medicine alone [18, 32]; one compared the adverse effects of Chinese medicine alone with that of Western medicine alone [29]; and four compared the adverse effects of the combined use of Chinese and Western medicine with that of either Chinese or Western medicine alone [17, 19, 21, 26]. Apart from one study [18], all reviews indicated fewer adverse effects in the experimental group than in the control group.

## 4. Discussion

### 4.1. Poor Methodological Quality of Systematic Reviews/Meta-Analyses of Using Chinese Medicine for the Treatment of PCOS

As systematic reviews/meta-analyses are an important source of evidence for guiding clinical decision-making in evidence-based medicine, they need to be strictly standardized. The low overall quality of the 18 reviews included in this study suggests that existing systematic reviews/meta-analyses of treatment with Chinese medicine for PCOS need to be improved and rigorously planned according to the PRISMA protocol. Moreover, the included reviews were neither registered nor provided a detailed exclusion list, which might have affected the accuracy of the results. Furthermore, failure to declare conflicts of interest makes it difficult to rule out potential conflicts, thereby affecting the objectivity of the review to some extent.

All the data and findings from the included studies suggested that the combined use of Chinese and Western medicine could improve the efficacy of PCOS treatment. However, due to the poor quality of the systematic reviews, the credibility of the results and evidence was compromised. Except for efficiency, which had a quality rating of moderate, all other evidence had a quality rating of low or very low. The analysis showed that ratings were reduced primarily because of methodological limitations and incorrect selection of the included studies. This was reflected mostly in the inadequate blinding, inappropriate randomization, wide 95% confidence intervals, and small sample sizes, which reduced the credibility of the conclusions.

With regard to the very low-quality evidence found in our analysis, an overview of systematic reviews of the use of acupuncture for the treatment of PCOS also generally resulted in low quality of evidence [35]. However, subsequent studies have confirmed that acupuncture does not support the treatment of PCOS [36]. While this does not directly imply that low-quality evidence must not be credible, examples suggest that low-quality evidence does contain the possibility of not supporting the treatment.

### 4.2. Suggestions for Future Systematic Reviews/Meta-Analyses of Chinese Medicine Treatment for PCOS

At present, letrozole and other drugs are considered first-line medications to treat PCOS, but their side effects and adverse reactions have been shown to reduce patient compliance [37]. Traditional Chinese medicine is becoming more and more widely used because of its milder side effects and adverse reactions and individual relevance [38]. Particularly, berberine not only improves symptoms but also reduces the risk of cardiovascular disease [39]. Meanwhile, the mechanism of traditional Chinese medicine in the treatment of PCOS needs to be further explored. Compared with letrozole, which has a standard of quantitative use, the use of traditional Chinese medicine is difficult to quantify. Different doctors may use different traditional Chinese medicines, and this difference may have a negative impact on the efficacy of the treatment. Despite this, Chinese medicine is a good way to treat PCOS.

It is recommended that future systematic reviews/meta-analyses of treatment with Chinese medicine for PCOS prepare a research plan in advance, including a literature exclusion list, and that they use the effect size in a reasonable manner and analyze sources of heterogeneity and biases carefully during the review. To minimize study limitations, correct randomization and appropriate blinding methods should be introduced as inclusion criteria. Moreover, although this study found that the quality rating for the evidence for efficiency was higher than that of other evidence, most articles did not specify how efficiency was evaluated. Therefore, it is suggested to unify the evaluation criteria of effective efficiency and choose more main indicators that can reflect the curative effect [40]. Furthermore, existing evidence does not support the advantage of Chinese medicine over Western medicine in aspects such as the ovulation rate. The fact that the center of the diamond of some studies was biased toward the control group indicated that the real results might even be that Western medicine is more effective. Therefore, it is recommended that subsequent research should carry out multicenter, large-sample RCTs, or factorial tests to verify the efficiency of Chinese medicine, thereby providing more reliable evidence for clinical guidance.

### 4.3. Study Limitations

This study utilized the AMSTAR2 tool and the GRADE system to review and evaluate the existing evidence for the treatment of PCOS with Chinese medicine. This study has the following limitations. First, only Chinese-language reviews of poor overall quality were included in this study, which may have resulted in biased and inaccurate results. Second, the AMSTAR2 tool and the GRADE system are highly subjective. Even with two evaluators, subjective factors or user error cannot be fully eliminated, and this can introduce biases and errors. Third, subgroup analysis was performed only for studies in which Western medicine was used in combination with Chinese medicine in the experimental groups, and no individual analysis of different types of Chinese medicine was carried out, thus making it difficult to identify the efficiencies of particular Chinese medicines or treatment theories.

## 5. Conclusions

At present, only low-quality evidence is available to suggest that combined treatment with Chinese and Western medicine is superior to Western medicine alone in improving the pregnancy rate, ovulation rate, serum hormone levels, and adverse effects of patients with PCOS. Future clinical trials and reviews of higher quality are recommended to clarify the efficacy of Chinese medicine and provide more accurate evidence.

## Figures and Tables

**Table 1:** Search strategy for databases.

Database	Search	Search strategy
PubMed	#1	((“Polycystic Ovary Syndrome” (MeSH term)) OR (Stein-Leventhal syndrome) OR (PCOS) OR (polycystic ovarian syndrome))
	#2	((“Meta-Analysis” (MeSH term)) OR (Meta-Analysis) OR (Systematic Reviews))
	#3	((“Medicine, Chinese Traditional” (MeSH term)) OR (Drugs, Chinese Herbal) OR (traditional Chinese medicine) OR (Integrative Medicine) OR (integrated Chinese and Western medicine) OR (Cinnamon) OR (Berberine) OR (Resveratrol) OR (Paeoniflorin) OR (Cryptotanshinone))
	#4	#1 AND #2 AND #3

CNKI	#1	SU = polycystic ovaries + polycystic ovary syndrome + polycystic ovarian syndrome + PCOS + “Stein-Leventhal syndrome”
	#2	SU = meta-analysis + systematic review
	#3	SU = Chinese medicine + Chinese herbs + Chinese herbal medicine + proprietary Chinese medicine + Chinese and Western medicine
	#4	#1 AND #2 AND #3

MeSH: medical subject headings, SU: subject.

**Table 2:** Characteristics of the systematic reviews included in this study.

Included systematic reviews	Number of databases searched	Number of studies included	Sample size	Experimental group	Control group	Evaluation tools
Yan Lun et al. 2015 [17]	4	13	1,148	Chinese medicine + Western medicine^#^	Western medicine	Cochrane risk of bias assessment tool
Xiao Chao 2016 [18]	7	12	1,213	Chinese medicine + Western medicine	Western medicine	Jadad
Li Nan et al. 2017 [19]	3	23	NA	Chinese medicine + Western medicine^#^	Western medicine	NA
Lu RuLing et al. 2018 [20]	7	22	1,676	Kidney tonifying herbs + Diane-35	Diane-35	Jadad
Xu LiFang et al. 2018 [21]	6	22	NA	Chinese medicine to tonify the kidneys and invigorate the blood + Western medicine^#^	Western medicine	Jadad
Xu Huayun et al. 2018 [22]	3	14	978	Herbal manual cycle + Western medicine^#^	Western medicine	Jadad
Huang Wenfang et al. 2018 [23]	5	14	1,057	Liver relaxation method + Western medicine	Western medicine	Cochrane risk of bias assessment tool
Liu Ying et al. 2019 [24]	5	11	1,128	Kuntai capsule + Western medicine	Western medicine	Cochrane risk of bias assessment tool
Yuan BoChao 2019 [25]	7	7	634	Chinese herbal remedies to tonify the kidneys and invigorate the blood	Clomiphene	Cochrane risk of bias assessment tool
Ji Lin 2019 [26]	7	34	NA	Chinese medicine to tonify the kidneys and invigorate the blood + Western medicine^#^	Western medicine	Cochrane risk of bias assessment tool
Xie Peng Peng et al. 2019 [27]	7	20	1,484	Plus or minus CangFu Guiphlegm Tang + Western medicine	Western medicine	Jadad
Zhong Yizheng et al. 2019 [28]	7	15	1,259	Compound Xuanju capsules + Western medicine	Western medicine	Jadad
Dong YuFang 2020 [29]	7	43	3,056	Chinese medicine	Western medicine	Cochrane risk of bias assessment tool
Li Nan et al. 2020 [30]	5	13	1,305	Kuntai capsule + letrozole	Letrozole	Jadad
Du Xiu et al. 2020 [31]	6	14	1,100	Compound Xuanju capsules + Western medicine	Western medicine	Cochrane risk of bias assessment tool
Lin BeiBei 2020 [32]	8	26	1,299	Chinese herbs + Western medicine for kidney and liver	Western medicine	Cochrane risk of bias assessment tool
Chen JinMing et al. 2020 [33]	5	7	502	Gueiren pills + Western medicine	Western medicine	Cochrane risk of bias assessment tool
Huang Ting et al. 2020 [34]	8	13	797	Kidney tonifying herbs + clomiphene	Clomiphene	Cochrane risk of bias assessment tool

*Note.* NA: not reported. ^#^The experimental group in the study used both Chinese medicine and a combination of Chinese and Western medicine.

**Table 3:** AMSTAR2 quality evaluation results (items 1–16).

Included systematic reviews	1	2	3	4	5	6	7	8	9	10	11	12	13	14	15	16	Credibility
Yan Lun et al. 2015 [17]	Y	N	N	Y	Y	N	N	PY	N	N	Y	NP	N	Y	Y	N	Very low
Xiao Chao 2016 [18]	Y	N	N	Y	Y	N	N	Y	Y	N	N	Y	Y	N	Y	N	Very low
Li Nan et al. 2017 [19]	Y	N	N	Y	Y	N	N	PY	N	N	N	NP	N	N	NP	N	Very low
Lu RuLing et al. 2018 [20]	Y	N	N	Y	Y	N	N	PY	Y	N	N	N	N	N	Y	N	Very low
Xu LiFang et al. 2018 [21]	Y	N	N	Y	N	N	N	PY	Y	N	N	N	N	Y	Y	N	Very low
Xu Huayun et al. 2018 [22]	Y	N	N	Y	Y	Y	N	PY	Y	N	N	Y	Y	N	Y	N	Very low
Huang Wenfang et al. 2018 [23]	Y	N	N	Y	Y	Y	N	PY	Y	N	Y	Y	Y	Y	Y	N	Very low
Liu Ying et al. 2019 [24]	Y	N	N	Y	Y	N	N	PY	Y	N	Y	NP	NP	Y	N	N	Very low
Yuan BoChao 2019 [25]	Y	N	N	Y	Y	Y	N	PY	Y	N	NP	Y	Y	N	N	N	Very low
Ji Lin 2019 [26]	Y	N	N	Y	Y	Y	N	PY	Y	N	Y	Y	Y	Y	Y	N	Very low
Xie Peng Peng et al. 2019 [27]	Y	N	N	Y	Y	Y	N	PY	Y	N	N	Y	Y	N	Y	N	Very low
ZhongYizheng et al. 2019 [28]	Y	N	N	Y	Y	Y	N	PY	Y	N	Y	Y	Y	Y	Y	N	Very low
Dong YuFang 2020 [29]	Y	N	N	Y	Y	Y	N	PY	Y	N	N	Y	Y	N	Y	N	Very low
Li Nan et al. 2020 [30]	Y	N	N	Y	Y	Y	N	PY	Y	N	Y	NP	N	Y	NP	N	Very low
Du Xiu et al. 2020 [31]	Y	N	N	Y	Y	Y	N	PY	Y	N	N	NP	N	N	Y	N	Very low
Lin BeiBei 2020 [32]	Y	N	N	Y	Y	Y	N	PY	Y	N	N	NP	Y	N	Y	N	Very low
Chen JinMing et al. 2020 [33]	Y	N	N	Y	Y	Y	N	PY	Y	N	Y	NP	N	Y	NP	N	Very low
Huang Ting et al. 2020 [34]	Y	N	N	Y	Y	Y	N	PY	Y	N	N	Y	Y	N	N	N	Very low
Percentage of reports	100	0	0	100	94	67	0	100	89	0	39	50	56	44	67	0	

*Note.* Item 1: did the research questions and inclusion criteria for the review include the components of PICO? Item 2: did the report of the review contain an explicit statement that the review methods were established prior to conducting the review, and did the report justify any significant deviations from the protocol? Item 3: did the review authors explain their selection of the study designs for inclusion in the review? Item 4: did the review authors use a comprehensive literature search strategy? Item 5: did the review authors perform study selection in duplicate? Item 6: did the review authors perform data extraction in duplicate? Item 7: did the review authors provide a list of excluded studies and justify the exclusions? Item 8: did the review authors describe the included studies in adequate detail? Item 9: did the review authors use a satisfactory technique to assess the risk of bias (RoB) in individual studies that were included in the review? Item 10: did the review authors report on the sources of funding for the studies included in the review? Item 11: if meta-analysis was performed did the review authors use appropriate methods for statistical combination of results? Item 12: if meta-analysis was performed, did the review authors assess the potential impact of RoB in individual studies on the results of the meta-analysis or other evidence synthesis? Item 13: did the review authors account for RoB in individual studies when interpreting/discussing the results of the review? Item 14: did the review authors provide a satisfactory explanation for, and discussion of, any heterogeneity observed in the results of the review? Item 15: if they performed quantitative synthesis, did the review authors carry out an adequate investigation of publication bias (small study bias) and discuss its likely impact on the results of the review? Item 16: did the review authors report any potential sources of conflict of interest, including any funding they received to conduct the review? *Y* = Yes; PY = Partially yes; *N* = No; NP = no meta-analysis performed.

**Table 4:** GRADE quality-of-evidence ratings for pregnancy rate.

Included systematic reviews	Number of studies included	Pregnancy rate effect (95% CI)	GRADE quality of evidence	Relegation factors	
Yan Lun et al. 2015 [17]	16	OR = 3.44, 95% CI (2.66, 4.43)	Low	①④
Xiao Chao 2016 [18]	9	RR = 1.91, 95% CI (1.59, 2.29)	Low	①⑤
Li Nan et al. 2017 [19]	12	OR = 2.96, 95% CI (2.35, 3.74)	Very low	①④⑤
Lu RuLing et al. 2018 [20]	8	OR = 3.34, 95% CI (2.23, 5.02)	Very low	①②④⑤
Xu LiFang et al. 2018 [21]	18	OR = 3.83, 95% CI (2.95, 4.96)	Very low	①④⑤
Xu Huayun et al. 2018 [22]	11	RR = 1.70, 95% CI (1.39, 2.09)	Moderate	①
Huang Wenfang et al. 2018 [23]	3	OR = 1.97, 95% CI (1.19, 3.25)	Very low	①④⑤
Liu Ying et al. 2019 [24]	11	RR = 1.71, 95% CI (1.46, 2.01)	Low	①⑤

*Note.* CI: confidence interval; OR: odds ratio; RR: relative risk; ①: limitation; ②: inconsistency; ③: indirectness; ④: publication bias; ⑤: imprecision.

**Table 5:** GRADE quality-of-evidence ratings for ovulation rate.

Included systematic reviews	Number of studies included	Ovulation rate effect (95% CI)	GRADE quality of evidence	Relegation factors
Yan Lun et al. 2015 [17]	9	OR = 2.18, 95% CI (1.63, 2.92)	Very low	①④⑤
Xiao Chao 2016 [18]	8	RR = 1.10, 95% CI (0.87, 1.39)	Very low	①②④⑤
Li Nan et al. 2017 [19]	6	OR = 2.70, 95% CI (1.32, 5.45)	Very low	①②④⑤
Huang Wenfang et al. 2018 [23]	6	OR = 2.18, 95% CI (1.77, 2.68)	Low	①⑤
Liu Ying et al. 2019 [24]	8	RR = 1.34, 95% CI (1.23, 1.46)	Low	①⑤
Yuan BoChao 2019 [25]	6	RR = 0.97, 95% CI (0.86, 1.09)	Very low	①②④⑤
Ji Lin 2019 [26]	14	OR = 1.92, 95% CI (1.40, 2.64)	Low	①②
Xie Peng Peng et al. 2019 [27]	10	RR = 1.17, 95% CI (1.02, 1.34)	Low	①④
Yuan BoChao 2019 [25]	5	RR = 1.18, 95% CI (1.03, 1.37)	Low	①④
Li Nan et al. 2020 [30]	4	OR = 3.91, 95% CI (1.95, 7.84)	Very low	①④⑤
Du Xiu et al. 2020 [31]	6	RR = 1.17, 95% CI (1.03, 1.34)	Very low	①②④
Lin BeiBei 2020 [32]	6	RR = 1.31, 95% CI (1.16, 1.48)	Low	①④
Chen JinMing et al. 2020 [33]	3	RR = 1.21, 95% CI (1.07, 1.37)	Very low	①④⑤

*Note.* CI: confidence interval; OR: odds ratio; RR: relative risk; ①: limitation; ②: inconsistency; ③: indirectness; ④: publication bias; ⑤: imprecision.

**Table 6:** GRADE quality-of-evidence ratings for efficiency.

Included systematic reviews	Number of studies included	Efficiency effect (95% CI)	GRADE quality of evidence	Relegation factors
Yan Lun et al. 2015 [17]	14	OR = 5.32, 95% CI (3.82, 7.41)	Low	①④
Xiao Chao 2016 [18]	7	RR = 1.27, 95% CI (1.19, 1.36)	Low	①⑤
Li Nan et al. 2017 [19]	8	OR = 3.90, 95% CI (2.92, 5.20)	Low	①④
Lu RuLing et al. 2018 [20]	11	OR = 4.22, 95% CI (2.86, 6.23)	Very low	①④⑤
Xu LiFang et al. 2018 [21]	18	OR = 2.83, 95% CI (2.06, 3.88)	Very low	①④⑤
Xu Huayun et al. 2018 [22]	13	RR = 1.19, 95% CI (0.87, 1.63)	Low	①⑤
Huang Wenfang et al. 2018 [23]	7	OR = 2.63, 95% CI (1.67, 4.15)	Very low	①④⑤
Ji Lin 2019 [24]	21	OR = 3.38, 95% CI (2.59, 4.41)	Moderate	①
Xie Peng Peng et al 2019 [27]	14	RR = 1.13, 95% CI (1.02, 1.24)	Moderate	①
ZhongYizheng et al. 2019 [28]	10	RR = 1.27, 95% CI (1.13, 1.44)	Moderate	①
Dong YuFang 2020 [29]	31	RR = 1.26, 95% CI (1.20, 1.32)	Moderate	①
Li Nan et al. 2020 [30]	4	OR = 3.42, 95% CI (1.76, 6.64)	Very low	①④⑤
Lin BeiBei 2020 [32]	18	RR = 1.26, 95% CI (1.17, 1.36)	Very low	①②④
Chen JinMing et al. 2020 [33]	6	RR = 1.26, 95% CI (1.15, 1.37)	Low	①④
Huang Ting et al. 2020 [34]	8	RR = 1.25, 95% CI (1.13, 1.37)	Very low	①④⑤

*Note.* CI: confidence interval; OR: odds ratio; RR: relative risk; ①: limitation; ②: inconsistency; ③: indirectness; ④: publication bias; ⑤: imprecision.

**Table 7:** GRADE quality-of-evidence ratings for testosterone level.

Included systematic reviews	Number of studies included	Testosterone effect (95% CI)	GRADE quality of evidence	Relegation factors
Xiao Chao 2016 [24]	8	SMD = –0.81, 95% CI (–1.46, –0.16)	Very low	①②④⑤
Yuan BoChao 2019 [25]	5	MD = –1.51, 95% CI (–1.64, –1.37)	Very low	①④⑤
Ji Lin 2019 [26]	24	SMD = –0.64, 95% CI (–0.97, –0.36)	Very low	①②⑤
Xie Peng Peng et al 2019 [27]	13	WMD = –0.93, 95% CI (–1.38, –0.28)	Very low	①②④⑤
ZhongYizheng et al. 2019 [28]	9	SMD = –1.59, 95% CI (–1.76, –1.41)	Very low	①②④
Dong YuFang 2020 [29]	37	SMD = –0.40, 95% CI (–0.65, –0.15)	Very low	①②④
Li Nan et al. 2020 [30]	3	SMD = –0.68, 95% CI (–3.99, 2.62)	Very low	①②④⑤
Du Xiu et al. 2020 [31]	5	RR = –0.53, 95% CI (–0.90, –0.16)	Very low	①②④
Lin BeiBei 2020 [32]	19	SMD = –0.20, 95% CI (–0.55, 0.16)	Very low	①②④⑤
Chen JinMing et al. 2020 [33]	2	MD = 0.95, 95% CI (0.15, 1.75)	Very low	①②④⑤

*Note.* CI: confidence interval; MD: mean difference; SMD: standardized mean difference; WMD: weighted mean difference; ①: limitation; ②: inconsistency; ③: indirectness; ④: publication bias; ⑤: imprecision.

**Table 8:** GRADE quality-of-evidence ratings for luteinizing hormone level.

Included systematic reviews	Number of studies included	Luteinizing hormone effect (95% CI)	GRADE quality of evidence	Relegation factors
Xiao Chao 2016 [18]	7	SMD = –1.16, 95% CI (–1.66, –0.66)	Very low	①②④⑤
Lu RuLing et al. 2018 [20]	18	MD = –1.84, 95% CI (–1.98, –1.70)	Very low	①②⑤
Yuan BoChao 2019 [25]	5	MD = –6.72, 95% CI (–7.32, –6.13)	Very low	①④⑤
Ji Lin 2019 [26]	23	SMD = –0.55, 95% CI (–0.74, –0.37)	Low	①④
Xie Peng Peng et al 2019 [27]	13	WMD = –0.95, 95% CI (–1.41, –0.52)	Very low	①②④
ZhongYizheng et al. 2019 [28]	9	SMD = –1.24, 95% CI (–1.39, –1.08)	Very low	①②④
Dong YuFang 2020 [29]	39	SMD = –0.38, 95% CI (–0.59, –0.16)	Very low	①②④
Li Nan et al. 2020 [30]	5	SMD = 1.67, 95% CI (–1.97, –1.37)	Very low	①②④
Lin BeiBei 2020 [32]	17	SMD = –0.78, 95% CI (–1.22, –0.34)	Very low	①②④
Chen JinMing et al. 2020 [33]	2	MD = 7.55, 95% CI (2.05, 13.04)	Very low	①②④⑤

*Note.* CI: confidence interval; MD: mean difference; SMD: standardized mean difference; WMD: weighted mean difference; ①: limitation; ②: inconsistency; ③: indirectness; ④: publication bias; ⑤: imprecision.

**Table 9:** GRADE quality-of-evidence ratings for follicle-stimulating hormone level.

Included systematic reviews	Number of studies included	Follicle-stimulating hormone effect (95% CI)	GRADE quality of evidence	Relegation factors
Ji Lin 2019 [26]	19	SMD = 0.12, 95% CI (–0.29, –0.53)	Very low	①②④
Xie Peng Peng et al 2019 [27]	11	WMD = –0.59, 95% CI (–0.98, –0.20)	Very low	①②④
ZhongYizheng et al. 2019 [28]	8	SMD = 0.66, 95% CI (0.51, 0.82)	Low	①④
Dong YuFang 2020 [29]	37	SMD = 0.01, 95% CI (–0.22, 0.25)	Very low	①②④⑤
Li Nan et al. 2020 [30]	5	SMD = –1.67, 95% CI (–3.05, –0.30)	Very low	①②④⑤
Chen JinMing et al. 2020 [33]	2	MD = 0.13, 95% CI (–0.39, 0.66)	Very low	①②④⑤

*Note.* CI: confidence interval; MD: mean difference; SMD: standardized mean difference; WM: weighted mean difference; ①: limitation; ②: inconsistency; ③: indirectness; ④: publication bias; ⑤: imprecision.

**Table 10:** GRADE quality-of-evidence ratings for luteinizing hormone/follicle-stimulating hormone level.

Included systematic reviews	Number of studies included	Luteinizing hormone/follicle-stimulating hormone effect (95% CI)	GRADE quality of evidence	Relegation factors
Xiao Chao 2016 [18]	4	MD = –0.81, 95% CI (–1.17, –0.45)	Very low	①②④⑤
Lu RuLing et al. 2018 [20]	12	MD = –0.25, 95% CI (–0.44, –0.06)	Very low	①②⑤
Ji Lin 2019 [26]	11	SMD = –0.45, 95% CI (–0.68, –0.23)	Low	①②
Xie Peng Peng et al. 2019 [27]	3	WMD = –1.04, 95% CI (–1.78, –0.33)	Very low	①②④⑤
Dong YuFang 2020 [29]	22	SMD = –0.39, 95% CI (–0.60, –0.19)	Very low	①②④
Lin BeiBei 2020 [32]	14	MD = –0.37, 95% CI (–0.53, –0.21)	Very low	①②④

*Note.* CI: confidence interval; MD: mean difference; SMD: standardized mean difference; WMD: weighted mean difference; ①: limitation; ②: inconsistency; ③: indirectness; ④: publication bias; ⑤: imprecision.

**Table 11:** GRADE quality-of-evidence ratings for adverse effects.

Included systematic reviews	Number of studies included	Adverse effects effect (95% CI)	GRADE quality of evidence	Relegation factors
Yan Lun et al. 2015 [17]	4	OR = 0.19, 95% CI (0.08, 0.46)	Low	①⑤
Xiao Chao 2016 [18]	4	RD = –0.05, 95% CI (–0.13, 0.03)	Very low	①②⑤
Li Nan et al. 2017 [19]	3	OR = 0.07, 95% CI (0.02, 0.23)	Very low	①②④⑤
Xu LiFang et al. 2018 [21]	10	OR = 0.26, 95% CI (0.09, 0.80)	Low	①⑤
Ji Lin 2019 [26]	13	OR = 0.26, 95% CI (0.12, 0.55)	Very low	①②⑤
Dong YuFang 2020 [29]	8	RR = 0.12, 95% CI (0.06, 0.25)	Low	①④
Lin BeiBei 2020 [32]	13	RR = 0.36, 95% CI (0.20, 0.63)	Low	①④

*Note.* CI: confidence interval; OR: odds ratio; RR: relative risk; ①: limitation; ②: inconsistency; ③: indirectness; ④: publication bias; ⑤: imprecision.

## Data Availability

The data used to support the finding of this study are stored in the FAIRDOMHub database (https://fairdomhub.org/projects/230) [41].

## Supplementary Materials

Supplementary Materials The supplementary materials include the extracted characteristics of each study, the score of each outcome, the AMSTAR2 quality evaluation results, and the GRADE quality-of-evidence ratings. These are the same data uploaded to FAIRDOMhub (https://fairdomhub.org/projects/230).
