# Supplementary material for: An Overview of Systematic Reviews of Using Chinese Medicine to Treat Polycystic Ovary Syndrome
Source: Evid Based Complement Alternat Med. 2021 May 28;2021:9935536. doi: 10.1155/2021/9935536 (PMC8179771; doi:10.1155/2021/9935536)
Supplement: Supplementary Materials — The supplementary materials include the extracted characteristics of each study, the score of each outcome, the AMSTAR2 quality evaluation results, and the GRADE quality-of-evidence ratings. These are the same data uploaded to FAIRDOMhub (https://fairdomhub.org/projects/230). [file 9935536.f1.pdf]

Sifting

Serial	outcomes	fund	database	num of study	Total	T	C	Bias evaluation
42	Relegation factors、Cycle ovulation rate、Improvement rate of menstruation、T、BMI		3+ChiCTR+ICTR+embase+void+CEN	4	280	Fu Fang Xuan Jv Jiao Nang+Western medicine	Western medicine	Cochranehandbook
40	efficiency、ovulation rate、Relegation factors		3+pbmd+Cochran	22	1763	traditional medicine+Western medicine	Western medicine	Cochranehandbook
39	efficiency、Relegation factors、ovulation rate、LH、LH/FSH、T		3+cbm	13	1148	traditional medicine+（Western medicine）	Western medicine	Cochranehandbook
35	efficiency、Relegation factors、ovulation rate、Cycle ovulation rate		3+cmb+pbmd+embase	12	1213	traditional medicine+Western medicine	Western medicine	Jadad
33	ovulation rate、Relegation factors		3	23	-	traditional medicine+（Western medicine）	Western medicine	-
32	Relegation factors、ovulation rate、efficiency		3	8	-	调周法+（Western medicine）	Western medicine	Jadad
30	总efficiency、androgen、LH、LH/FSH、Relegation factors	Guangdong Provincial Science and Technology Planning Project（2014A020212273）;	3+cbm+pbmd+co+sci	22	1676	Tonify the kidneytraditional medicine+Diane-35	Diane-35	Jadad
28	efficiency、Relegation factors	Chinese medicine project of Jiangxi Health and Family Planning Commission【No.2015A087】;	3+pbmd+medline+Sprin	22	-	traditional medicineTonify the kidneyInvigorate the	Western medicine	Jadad
22	efficiency、Relegation factors、Cycle ovulation rate	Medical Guidance Science and technology supporting project of Shanghai Science and Technology Commission（16401934500）；Shanghai School of Traditional Chinese medicine cai gynecological school heritage	3	14	978	traditional medicine人工Cycle +（Western medicine）	Western medicine	Jadad
21	efficiency、ovulation rate、Cycle ovulation rate、Relegation factors	Surface Project of National Natural Science Foundation of China（No:81673661）;	3+pbmd+Cochran	14	1057	舒肝法+Western medicine	Western medicine	Cochranehandbook
20	Relegation factors、ovulation rate		3+pbmd+ovid	11	1128	KunTai capsule+Western medicine	Western medicine	Cochranehandbook
19	Relegation factors、ovulation rate、FSH、LH、T、Insulin content、		3+cbm+3	7	634	traditional medicineTonify the kidneyInvigorate the [illegible]	Clomifene Citrate	Cochranehandbook
17	efficiency、Relegation factors、ovulation rate、Ovarian volume、Number of mature follicles、LH、FSH、LH/FSH、E2、T		3+cbm+2+medline	34	-	traditional medicineTonify the kidneyInvigorate the blood法+Western	Western medicine	Cochranehandbook
13	efficiency、FSH、LH、LH/FSH、T、BMI、ovulation rate、physical sign score	Projects supported by the National Natural Science Foundation of China（No.81804135）;	3+cbm+3	20	1484	Cang Fu Dao Tan Tang+Western medicine	Western medicine	Jadad
12	efficiency、ovulation rate、Relegation factors、estrogen、Degree of endometrial thickening	Surface Project of National Natural Science Foundation of China（No.81801466）;	3+cbm+3	15	1259	Fu Fang Xuan Jv Jiao Nang+Western medicine	Western medicine	Jadad
11	efficiency、TCM syndrome、Relegation factors、Biphasic rate of basal body temperature、TCM syndrome socer、BMI、Acne score、LH、FSH、LH/FSH、T、E2、PRL、Insulin content、etc.		3+cbm+3	43	3056	traditional medicine	Western medicine	Cochranehandbook
10	efficiency、ovulation rate、Relegation factors、Number of mature follicles、Endometrial thickness、Cervical mucus score、FSH、LH、PRL、E2、T	National Natural Science Foundation of China（No:81802630）	3+cmb+1	13	1305	KunTai capsule+Letrozole	Letrozole	Jadad
8	Relegation factors、Cycle ovulation rate、TCM syndrome、T、Endometrial thickness	Guangxi University of Chinese Medicine school-level research program（NO.2016MS009）;State Administration of Traditional Chinese Medicine	3+Chi+pbmd+c	14	1100	Fu Fang Xuan Jv Jiao Nang+Western medicine	Western medicine	Cochranehandbook
7	efficiency、ovulation rate、Relegation factors、T、LH、LH/FSH		3+cbm+3+1	26	1299	traditional medicine+Western	Western medicine	Cochranehandbook
4	efficiency、Relegation factors、ovulation rate、Ovarian volume、T、LH、FSH	Natural Science Foundation of Fujian Province（NO.2019J01351）;Key projects supported by the Joint Fund of the National Natural Science Foundation of China（NO.[illegible]）	3+pbmd+Cochran	7	502	Gui Shen Wan+Western medicine	Western medicine	Cochranehandbook
2	Endometrial thickness, endometrial type, uterine a pulsation index, uterine a resistance index、Relegation factorsTCM syndrome score、efficiency	State Administration of Traditional Chinese Medicine（1199ws02）Subject of Guangdong Traditional Chinese Medicine Bureau（20181120）	3+1+3+1	13	797	traditional medicine+Western medicine	Western medicine	CochraneCollaborationtools

Characteristics

Serial number	Included Systematic Reviews	Number of databases	number of Include RCTs	Total subjects	Experimental group	Control group	Bias evaluation	fund	conclusion	备注	库数
40	Yan, Lun et al. 2015	4	13	1148	Chinese medicine + (Western medicine)	Western medicine	Cochranehandbook		Tonify the kidneytraditional medicine联合CC治疗PCOS的临床效果高于单纯CC治疗PCOS的临床效果，其缓解率、ovulation rate、Relegation factors均有提高，并可降low未破裂卵泡黄素化综合征的发生率。但Tonify the kidneytraditional medicine治疗PCOS的机制尚未完全明确，有待进一步SR。		3+cbm
39	Xiao Chao 2016	7	12	1213	Chinese Medicine + Western Medicine	Western medicine	Jadad		肾Invigorate the blood法或联合促排卵药对提高PCOS患者的总efficiency、Relegation factors，改善血清LH、LH/FSH、T水平，均有较好疗效;但没有足够的统计学证据证明Tonify the kidneyInvigorate the blood法或联合促排卵药在改善排卵方面的效果优于单纯使用促排卵药;Tonify the kidneyInvigorate the blood法治疗PCOS的安全性也有待进一步讨论。	ovulation rate Not statistically significant	3+cmb+pbmd+embase
35	Li Nan et al. 2017	3	23	-	Chinese medicine + (Western medicine)	Western medicine	-		中西医结合治疗PCOS不孕症疗效较好，是一种具有发展前景的治疗方法。		3
30	Lu Ru-Ling et al. 2018	7	22	1676	Kidney Tonic Herbs + Daing-35	Daying-35	Jadad	Guangdong Provincial Science and Technology Planning Project（2014A020212273）；	现有证据显示Tonify the kidneytraditional medicine联合Diane-35治疗PCOS疗效显著，能明显改善性激素水平，提高总efficiency及Relegation factors，adverse effects少，可供临床参考与应用。		3+cbm+pbmd+co+sci
28	Xu, Li-Fang et al. 2018	6	22	-	Chinese medicine to tonify the kidneys and invigorate the blood + (Western medicine)	Western medicine	Jadad	Chinese medicine project of Jiangxi Health and Family Planning Commission【No.2015A087】；	Tonify the kidneyInvigorate the bloodtraditional medicine联合Western medicine治疗多囊卵巢综合征在改善临床症状、physical sign及妊娠方面明显优于单用Western medicine治疗，且中Western medicine联合应用安全性要明显高于Western medicine单用;而Tonify the kidneyInvigorate the bloodtraditional medicine单用于多囊卵巢综合征改善临床症状及physical sign上与Western medicine比较无明显优势，但在Relegation factors上有明显优势，但是由于纳入文献数量较少且文献质量 score较low，其有效性		3+pbmd+medline+SpringerLink
22	Xu Huayun et al. 2018	3	14	978	Herbal manual cycle + (Western)	Western medicine	Jadad	Medical Guidance Science and technology supporting project of Shanghai Science and Technology Commission（16401934500）；Shanghai School of Traditional Chinese medicine cai gynecological school heritage SR base（ZY3-CCCX-1-1006）；	traditional medicine人工Cycle 治疗PCOS的疗效肯定，在efficiency、Relegation factors、Cycle ovulation rate方面优于单用Western medicine治疗，且未见明显adverse effects，但纳入的临床试验数量有限，在随机化、adverse effects报道等方面存在缺陷，建议进一步开展多中心、大样本、高质量的随机对照试验，并对adverse effects做充分的SR和报道。		3
21	Huang, Wenfang et al. 2018	5	14	1057	Liver relaxation method + Western medicine	Western medicine	Cochranehandbook	Surface Project of National Natural Science Foundation of China（No:81673661）；	运用疏肝法对肝郁型多囊卵巢综合征治疗后临床总efficiency以及ovulation rate、Cycle ovulation rate、Relegation factors均有显著提高，且效果优于Western medicine对照组，说明运用中医辨证治疗PCOS患者尤其要重视肝郁症，对临床治疗多囊卵巢综合征具有指导意义。		3+pbmd+Cochran
20	Liu Ying et al. 2019	5	11	1128	Kuntai capsule + Western medicine	Western medicine	Cochranehandbook		KunTai capsule联合用药能有效提高Relegation factors及ovulation rate，但要将其推广应用，尚需大样本、多中心的随机对照临床试验进一步证实。		3+pbmd+ovid
19	Hara Bocchio 2019	7	7	634	Chinese herbal remedies to tonify the kidneys and invigorate the blood	clomiphene	Cochranehandbook		Tonify the kidneyInvigorate the bloodtraditional medicine治疗多囊卵巢综合征导致不孕症临床疗效较好，安全性较高，但由于纳入文献SR质量相对偏low，结果可能存在偏倚，还需要更多高质量的临床研究加以验证。	ovulation rate Not statistically significant	3+cbm+3
17	Ji Lin 2019	7	34	-	Chinese medicine to tonify the kidneys and invigorate the blood + (Western medicine)	Western medicine	Cochranehandbook		表明了Tonify the kidneyInvigorate the blood法治疗多囊卵巢综合征的疗效和安全性，为Tonify the kidneyInvigorate the blood法治疗多囊卵巢综合征提供了证据。	FSH Not statistically significant	3+cbm+2+medline
13	Xie Peng Peng et al. 2019	7	20	1484	Plus or minus Cang Fu Gui Phlegm Tang + Western medicine	Western medicine	Jadad	Projects supported by the National Natural Science Foundation of China（No.81804135）；	对比单用化学药治疗PCOS，加减苍附导痰汤联合化学药可提高总efficiency和ovulation rate，降low血清激素水平、BMI指数及physical sign score。		3+cbm+3
12	Zhong Yizheng et al. 2019	7	15	1259	Compound Xuanju Capsules + Western Medicine	Western medicine	Jadad	Surface Project of National Natural Science Foundation of China（No.81801466）；	Fu Fang Xuan Jv Jiao Nang联合化学药对比单用化学药治疗PCOS在提高总efficiency、ovulation rate、Relegation factors、子宫内膜增厚程度，降low血清estrogen水平方面均优于单用化学药。		3+cbm+3
11	Yufang Dong 2020	7	43	3056	Chinese medicine	Western medicine	Cochranehandbook		中医药组治疗多囊卵巢综合征的临床疗效总efficiency较Western medicine组好;中医药治疗能够提高临床治愈率、显效率，TCM syndrome疗效总efficiency、显效率，降lowTCM syndrome积分，促进正常月经恢复，进一步说明中医药治疗多囊卵巢综合征临床疗效确切。	FSH Not statistically significant	3+cbm+3
10	Li Nan et al. 2020	5	13	1305	Kuntai capsule + Letrozole	letrozole (antibiotic)	Jadad	National Natural Science Foundation of China（No:81802630）	KunTai capsule联合Letrozole治疗PCOS的效果优于单药Letrozole治疗，可促进排卵，最终提高患者Relegation factors。但由于纳入试验的数量和质量原因，联合治疗的推广仍需更多高质量的临床随机试验的验证。		3+cmb+1
8	Du Xiu et al. 2020	6	14	1100	Compound Xuanju Capsules + Western Medicine	Western medicine	Cochranehandbook	Guangxi University of Chinese Medicine school-level research program（NO.2016MS009）;State Administration of Traditional Chinese Medicine	Fu Fang Xuan Jv Jiao Nang是治疗多囊卵巢综合征不孕症的有效药物。但尚需更多大样本高质量临床试验予以验证。		3+Chi+pbmd+c
7	Lim Bui Bui 2020	8	26	1299	Chinese herbs + Western medicine for kidney and liver	Western medicine	Cochranehandbook		Tonify the kidney疏肝法治疗多囊卵巢综合征具有较好的临床疗效和安全性。		3+cbm+3+1
4	Chan Kam Ming et al. 2020	5	7	502	Guei Ren Pills + Western Medicine	Western medicine	Cochranehandbook	Natural Science Foundation of Fujian Province（NO.2019J01351）;Key projects supported by the Joint Fund of the National Natural Science Foundation of China	与常规Western medicine相比，归肾丸联合常规Western medicine在PCOS的治疗中能显著改善患者的Relegation factors、ovulation rate、Ovarian volume和性激素水平，且安全性良好。	FSH Not statistically significant	3+pbmd+Cochran
2	Huang Ting et al. 2020	8	13	797	Kidney Tonic Herbs + Clomiphene	clomiphene	Cochranehandbook	State Administration of Traditional Chinese Medicine（1199ws02）Subject of Guangdong Traditional Chinese Medicine Bureau（20181120）	对比单纯使用克罗米芬，Tonify the kidneytraditional medicine联合克罗米芬能有效促进PCOS患者的子宫内膜增长，并改善其子宫内膜类型，促进子宫内膜血流灌注，提高Relegation factors，同时改善临床症状，降lowTCM syndrome score，提高治疗总efficiency。		3+1+3+1

表格 1

Serial number	Included Systematic Reviews	Experimental group	Control group	Relegation factors	Relegation factorsGRADE quality of evidence	Relegation factorsRelegation factors	efficiency	efficiencyGRADE quality of evidence	efficiencyRelegation factors	ovulation rate	ovulation rateGRADE quality of evidence	ovulation rateRelegation factors	T	T	T	LH	LH	LH	FSH	FSH	FSH	L/F	L/F	L/F	adverse effects	conclusion	备注
40	Yan, Lun et al. 2015	Chinese medicine + (Western medicine)	Western medicine	OR=3.44，95%CI(2.66，4.43)	low16	①④	OR=5.32，95%CI(3.82，7.41)	low14	①④	OR=2.18，95%CI(1.63，2.92)	very low9	①④⑤	-	-	-	-	-	-	-	-	-	-	-	-	OR=0.19，95%CI(0.08，0.46)low4①⑤	Tonify the kidneytraditional medicine联合CC治疗PCOS的临床效果高于单纯CC治疗PCOS的临床效果，其缓解率、ovulation rate、Relegation factors均有提高，并可降low未破裂卵泡黄素化综合征的发生率。但Tonify the kidneytraditional medicine治疗PCOS的机制尚未完全明确，有待进一步SR。	
39	Xiao Chao 2016	Chinese Medicine + Western Medicine	Western medicine	RR=1.91，95%CI(1.59，2.29)	low9	①⑤	RR=1.27，95%CI(1.19，1.36)	low7	①⑤	RR=1.10，95%CI(0.87，1.39)	very low8	①②④⑤	SMD=-0.81，95%CI(-1.46，-0.16)	very low8	①②④⑤	SMD=-1.16，95%CI(-1.66，-0.66)	very low7	①②④⑤	-	-	-	MD=-0.81，95%CI(-1.17，-0.45)	very low4	①②④⑤	RD=-0.05，95%CI(-0.13，0.03)very low4①②⑤	肾Invigorate the blood法或联合促排卵药对提高PCOS患者的总efficiency、Relegation factors，改善血清LH、LH/FSH、T水平，均有较好疗效;但没有足够的统计学证据证明Tonify the kidneyInvigorate the blood法或联合促排卵药在改善排卵方面的效果优于单纯使用促排卵药;Tonify the kidneyInvigorate the blood法治疗PCOS的安全性也有待进一步讨论。	ovulation rate Not statistically significant
35	Li Nan et al. 2017	Chinese medicine + (Western medicine)	Western medicine	OR=2.96，95%CI(2.35，3.74)	very low12	①④⑤	OR=3.90，95%CI(2.92，5.20)	low8	①④	OR=2.70，95%CI(1.32，5.45)	very low6	①②④⑤	-	-	-	-	-	-	-	-	-	-	-	-	OR=0.07，95%CI(0.02，0.23)very low3①②④⑤	中西医结合治疗PCOS不孕症疗效较好，是一种具有发展前景的治疗方法。	
30	Lu Ru-Ling et al. 2018	Kidney Tonic Herbs + Daing-35	Daying-35	OR=3.34，95%CI(2.23，5.02)	very low8	①②④⑤	OR=4.22，95%CI(2.86，6.23)	very low11	①④⑤	-	-	-	-	-	-	MD=-1.84，95%CI(-1.98，-1.70)	very low18	①②⑤	-	-	-	MD=-0.25，95%CI(-0.44，-0.06)	very low12	①②⑤		现有证据显示Tonify the kidneytraditional medicine联合Diane-35治疗PCOS疗效显著，能明显改善性激素水平，提高总efficiency及Relegation factors，adverse effects少，可供临床参考与应用。	
28	Xu, Li-Fang et al. 2018	Chinese medicine to tonify the kidneys and invigorate the blood + (Western medicine)	Western medicine	OR=3.83，95%CI(2.95，4.96)	very low18	①④⑤	OR=2.83，95%CI(2.06，3.88)	very low18	①④⑤	-	-	-	-	-	-	-	-	-	-	-	-	-	-	-	OR=0.26，95%CI(0.09，0.80)low10①⑤	Tonify the kidneyInvigorate the bloodtraditional medicine联合Western medicine治疗多囊卵巢综合征在改善临床症状、physical sign及妊娠方面明显优于单用Western medicine治疗，且中Western medicine联合应用安全性要明显高于Western medicine单用;而Tonify the kidneyInvigorate the bloodtraditional medicine单用于多囊卵巢综合征改善临床症状及physical sign上与Western medicine比较无明显优势，但在Relegation factors上有明显优势，但是由于included RCTs量较少且	
22	Xu Huayun et al. 2018	Herbal manual cycle + (Western)	Western medicine	RR=1.70，95%CI(1.39，2.09)	Moderate11	①	RR=1.19，95%CI(0.87，1.63)	low13	①⑤	-	-	-	-	-	-	-	-	-	-	-	-	-	-	-		traditional medicine人工Cycle 治疗PCOS的疗效肯定，在efficiency、Relegation factors、Cycle ovulation rate方面优于单用Western medicine治疗，且未见明显adverse effects。但纳入的临床试验数量有限，在随机化、adverse effects报道等方面存在缺陷，建议进一步开展多中心、大样本、高质量的随机对照试验，并对adverse effects做充分的SR和报道。	
21	Huang, Wenfang et al. 2018	Liver relaxation method + Western medicine	Western medicine	OR=1.97，95%CI(1.19，3.25)	very low3	①④⑤	OR=2.63，95%CI(1.67，4.15)	very low7	①④⑤	OR=2.18，95%CI(1.77，2.68)	low6	①⑤	-	-	-	-	-	-	-	-	-	-	-	-		运用疏肝法对肝郁型多囊卵巢综合征治疗后临床总efficiency以及ovulation rate、Cycle ovulation rate、Relegation factors均有显著提高，且效果优于Western medicine对照组，说明运用中医辨证治疗PCOS患者尤其要重视肝郁症，对临床治疗多囊卵巢综合征具有指导意义。	
20	Liu Ying et al. 2019	Kuntai capsule + Western medicine	Western medicine	RR=1.71，95%CI(1.46，2.01)	low11	①⑤	-	-	-	RR=1.34，95%CI(1.23，1.46)	low8	①⑤	-	-	-	-	-	-	-	-	-	-	-	-		KunTai capsule联合用药能有效提高Relegation factors及ovulation rate，但要将其推广应用，尚需大样本、多中心的随机对照临床试验进一步证实。	
19	Hara Bocchio 2019	Chinese herbal remedies to tonify the kidneys and invigorate the blood	clomiphene	RR=1.78，95%CI(1.44，2.19)	low7	①⑤	-	-	-	RR=0.97，95%CI(0.86，1.09)	very low6	①②④⑤	MD=-1.51，95%CI(-1.64，-1.37)	very low5	①④⑤	MD=-6.72，95%CI(-7.32，-6.13)	very low5	①④⑤	-	-	-	-	-	-		Tonify the kidneyInvigorate the bloodtraditional medicine治疗多囊卵巢综合征导致不孕症临床疗效较好，安全性较高，但由于纳入文献SR质量相对偏low，结果可能存在偏倚，还需要更多高质量的临床研究加以验证。	ovulation rate Not statistically significant
17	Ji Lin 2019	Chinese medicine to tonify the kidneys and invigorate the blood + (Western medicine)	Western medicine	OR=2.78，95%CI(2.21，3.51)	low17	①④	OR=3.38，95%CI(2.59，4.41)	Moderate	①	OR=1.92，95%CI(1.40，2.64)	low14	①②	SMD=-0.64，95%CI(-0.97，-0.36)	very low24	①②⑤	SMD=-0.55，95%CI(-0.74，-0.37)	low23	①④	SMD=0.12，95%CI(-0.29，-0.53)	very low19	①②④	SMD=-0.45，95%CI(-0.68，-0.23)	low11	①②	OR=0.26，95%CI(0.12，0.55)very low13①②⑤	表明了Tonify the kidneyInvigorate the blood法治疗多囊卵巢综合征的疗效和安全性，为Tonify the kidneyInvigorate the blood法治疗多囊卵巢综合征提供了证据。	FSH Not statistically significant
13	Xie Peng Peng et al. 2019	Plus or minus Cang Fu Gui Phlegm Tang + Western medicine	Western medicine	-	-	-	RR=1.13，95%CI(1.02，1.24)	Moderate	①	RR=1.17，95%CI(1.02，1.34)	low10	①④	WMD=-0.93，95%CI(-1.38，-0.28)	very low13	①②④⑤	WMD=-0.95，95%CI(-1.41，-0.52)	very low13	①②④	WMD=-0.59，95%CI(-0.98，-0.20)	very low11	①②④	WMD=-1.04，95%CI(-1.78，-0.33)	very low3	①②④⑤		对比单用化学药治疗PCOS，加减苍附导痰汤联合化学药可提高总efficiency和ovulation rate，降low血清激素水平、BMI指数及physical sign score。	
12	Zhong Yizheng et al. 2019	Compound Xuanju Capsules + Western Medicine	Western medicine	RR=1.34，95%CI(1.11，1.61)	low6	①④	RR=1.27，95%CI(1.13，1.44)	Moderate	①	RR=1.18，95%CI(1.03，1.37)	low5	①④	SMD=-1.59，95%CI(-1.76，-1.41)	very low9	①②④	SMD=-1.24，95%CI(I-1.39，-1.08)	very low9	①②④	SMD=0.66，95%CI(0.51，0.82)	low8	①④	-	-	-		Fu Fang Xuan Jv Jiao Nang联合化学药对比单用化学药治疗PCOS在提高总efficiency、ovulation rate、Relegation factors、子宫内膜增厚程度，降low血清estrogen水平方面均优于单用化学药。	
11	Yufang Dong 2020	Chinese medicine	Western medicine	RR=1.94，95%CI(1.14，3.29)	very low8	①④⑤	RR=1.26，95%CI(1.20，1.32)	Moderate	①	-	-	-	SMD=-0.40，95%CI(-0.65，-0.15)	very low37	①②④	SMD=-0.38，95%CI(-0.59，-0.16)	very low39	①②④	SMD=0.01，95%CI(-0.22，0.25)	very low37	①②④⑤	SMD=-0.39，95%CI(-0.60，-0.19)	very low22	①②④	RR=0.12，95%CI(0.06，0.25)low8①④	中医药组治疗多囊卵巢综合征的临床疗效总efficiency较Western medicine组好;中医药治疗能够提高临床治愈率、显效率，TCM syndrome疗效总efficiency、显效率，降lowTCM syndrome积分，促进正常月经恢复，进一步说明中医药治疗多囊卵巢综合征临床疗效确切。	FSH Not statistically significant
10	Li Nan et al. 2020	Kuntai capsule + Letrozole	letrozole (antibiotic)	OR=2.49，95%CI(1.79，3.45)	very low8	①④⑤	OR=3.42，95%CI(1.76，6.64)	very low4	①④⑤	OR=3.91，95%CI(1.95，7.84)	very low4	①④⑤	SMD=-0.68，95%CI(-3.99，2.62)	very low3	①②④⑤	SMD=1.67，95%CI(-1.97，-1.37)	very low5	①②④	SMD=-1.67，95%CI(-3.05，-0.30)	very low5	①②④⑤	-	-	-		KunTai capsule联合Letrozole治疗PCOS的效果优于单药Letrozole治疗，可促进排卵，最终提高患者Relegation factors。但由于纳入试验的数量和质量原因，联合治疗的推广仍需更多高质量的临床随机试验的验证。	
8	Du Xiu et al. 2020	Compound Xuanju Capsules + Western Medicine	Western medicine	RR=1.68，95%CI(1.45，1.94)	Moderate14	①	-	-	-	RR=1.17，95%CI(1.03，1.34)	very low6	①②④	RR=-0.53，95%CI(-0.90，-0.16)	very low5	①②④	-	-	-	-	-	-	-	-	-		Fu Fang Xuan Jv Jiao Nang是治疗多囊卵巢综合征不孕症的有效药物。但尚需更多大样本高质量临床试验予以验证。	
7	Lim Bui Bui 2020	Chinese herbs + Western medicine for kidney and liver	Western medicine	RR=1.69，95%CI(1.45，1.98)	low14	①④	RR=1.26，95%CI(1.17，1.36)	very low18	①②④	RR=1.31，95%CI(1.16，1.48)	low6	①④	SMD=-0.20，95%CI(-0.55，0.16)	very low19	①②④⑤	SMD=-0.78，95%CI(-1.22，-0.34)	very low17	①②④	-	-	-	MD=-0.37，95%CI(-0.53，-0.21)	very low14	①②④	RR=0.36，95%CI(0.20，0.63)low13①④	Tonify the kidney疏肝法治疗多囊卵巢综合征具有较好的临床疗效和安全性。	
4	Chan Kam Ming et al. 2020	Guei Ren Pills + Western Medicine	Western medicine	RR=1.56，95%CI(1.27，1.91)	low6	①④	RR=1.26，95%CI(1.15，1.37)	low6	①④	RR=1.21，95%CI(1.07，1.37)	very low3	①④⑤	MD=0.95，95%CI(0.15，1.75)	very low2	①②④⑤	MD=7.55，95%CI(2.05，13.04)	very low2	①②④⑤	MD=0.13，95%CI(-0.39，0.66)	very low2	①②④⑤	-	-	-		与常规Western medicine相比，归肾丸联合常规Western medicine在PCOS的治疗中能显著改善患者的Relegation factors、ovulation rate、Ovarian volume和性激素水平，且安全性良好。	FSH Not statistically significant
2	Huang Ting et al. 2020	Kidney Tonic Herbs + Clomiphene	clomiphene	RR=2.18，95%CI(1.55，3.05)	very low8	①④⑤	RR=1.25;95%CI(1.13，1.37)	very low8	①④⑤	-	-	-	-	-	-	-	-	-	-	-	-	-	-	-	Tonify the kidneytraditional medicine联合克罗米芬组中出现3例口干、4例头晕，克罗米芬组中出现2例头痛、2例便秘、1例皮疹、1例卵巢过度刺激综合征、1例脱发、1例未破裂卵泡黄素化综合征	对比单纯使用克罗米芬，Tonify the kidneytraditional medicine联合克罗米芬能有效促进PCOS患者的子宫内膜增长，并改善其子宫内膜类型，促进子宫内膜血流灌注，提高Relegation factors，同时改善临床症状，降lowTCM syndrome score，提高治疗总efficiency。	

① Limitation; ② Inconsistency; ③ Indirectness; ④ Publication bias; ⑤ Imprecision.

AMSTAR 2

	Included Systematic Reviews	1	2	3	4	5	6	7	8	9	10	11	12	13	14	15	16	Credibility	
40	Yan, Lun et al. 2015	Y	N	N	Y	Y	N	N	PY	N	N	Y	NP	N	Y	Y	N	Very low	
39	Xiao Chao 2016	Y	N	N	Y	Y	N	N	Y	Y	N	N	Y	Y	N	Y	N	Very low	
35	Li Nan et al. 2017	Y	N	N	Y	Y	N	N	PY	N	N	N	NP	N	N	NP	N	Very low	
30	Ruling Lu et al. 2018	Y	N	N	Y	Y	N	N	PY	Y	N	N	N	N	N	Y	N	Very low	
28	Xu, Li-Fang et al. 2018	Y	N	N	Y	N	N	N	PY	Y	N	N	N	N	Y	Y	N	Very low	
22	Xu Huayun et al. 2018	Y	N	N	Y	Y	Y	N	PY	Y	N	N	Y	Y	N	Y	N	Very low	
21	Huang, Wenfang et al. 2018	Y	N	N	Y	Y	Y	N	PY	Y	N	Y	Y	Y	Y	Y	N	Very low	
20	Liu Ying et al. 2019	Y	N	N	Y	Y	N	N	PY	Y	N	Y	NP	NP	Y	N	N	Very low	
19	Original Bochao 2019	Y	N	N	Y	Y	Y	N	PY	Y	N	NP	Y	Y	N	N	N	Very low	
17	Jilin 2019	Y	N	N	Y	Y	Y	N	PY	Y	N	Y	Y	Y	Y	Y	N	Very low	
13	Xie Peng Peng et al 2019	Y	N	N	Y	Y	Y	N	PY	Y	N	N	Y	Y	N	Y	N	Very low	
12	Zhong Yizheng et al. 2019	Y	N	N	Y	Y	Y	N	PY	Y	N	Y	Y	Y	Y	Y	N	Very low	
11	Tung Yu Fong 2020	Y	N	N	Y	Y	Y	N	PY	Y	N	N	Y	Y	N	Y	N	Very low	
10	Li Nan et al. 2020	Y	N	N	Y	Y	Y	N	PY	Y	N	Y	NP	N	Y	NP	N	Very low	
8	Du Xiu et al. 2020	Y	N	N	Y	Y	Y	N	PY	Y	N	N	NP	N	N	Y	N	Very low	
7	Limpibe 2020	Y	N	N	Y	Y	Y	N	PY	Y	N	N	NP	Y	N	Y	N	Very low	
4	Chan Kam Ming 2020	Y	N	N	Y	Y	Y	N	PY	Y	N	Y	NP	N	Y	NP	N	Very low	
2	Wong Ting Ting 2020	Y	N	N	Y	Y	Y	N	PY	Y	N	N	Y	Y	N	N	N	Very low	
	Percentage of reports	100%	0%	0%	100%	94%	67%	0%	100%	89%	0%	39%	50.0%	56%	44%	67%	0%		

pregnancy rate

	SR	include RCTs	pregnancy rate	GRADE quality of evidence	Relegation factors
40	Yan, Lun et al. 2015	16	OR=3.44，95%CI(2.66，4.43)	low	①④
39	Xiao Chao 2016	9	RR=1.91，95%CI(1.59，2.29)	low	①⑤
35	Li Nan et al. 2017	12	OR=2.96，95%CI(2.35，3.74)	very low	①④⑤
30	Ruling Lu et al. 2018	8	OR=3.34，95%CI(2.23，5.02)	very low	①②④⑤
28	Xu, Li-Fang et al. 2018	18	OR=3.83，95%CI(2.95，4.96)	very low	①④⑤
22	Xu Huayun et al. 2018	11	RR=1.70，95%CI(1.39，2.09)	Moderate	①
21	Huang, Wenfang et al. 2018	3	OR=1.97，95%CI(1.19，3.25)	very low	①④⑤
20	Liu Ying et al. 2019	11	RR=1.71，95%CI(1.46，2.01)	low	①⑤
19	Original Bochao 2019	7	RR=1.78，95%CI(1.44，2.19)	low	①⑤
17	Jilin 2019	17	OR=2.78，95%CI(2.21，3.51)	low	①④
12	Zhong Yizheng et al. 2019	6	RR=1.34，95%CI(1.11，1.61)	low	①④
11	Tung Yu Fong 2020	8	RR=1.94，95%CI(1.14，3.29)	very low	①④⑤
10	Li Nan et al. 2020	8	OR=2.49，95%CI(1.79，3.45)	very low	①④⑤
8	Du Xiu et al. 2020	14	RR=1.68，95%CI(1.45，1.94)	Moderate	①
7	Limpibe 2020	14	RR=1.69，95%CI(1.45，1.98)	low	①④
4	Chan Kam Ming et al. 2020	6	RR=1.56，95%CI(1.27，1.91)	low	①④
2	Huang Ting et al. 2020	8	RR=2.18，95%CI(1.55，3.05)	very low	①④⑤

① Limitation; ② Inconsistency; ③ Indirectness;
④ Publication bias; ⑤ Imprecision.

efficiency

	SR	include RCTs	efficiency	GRADE quality of evidence	Relegation factors
40	Yan, Lun et al. 2015	14	OR=5.32，95%CI(3.82，7.41)	low	①④
39	Xiao Chao 2016	7	RR=1.27，95%CI(1.19，1.36)	low	①⑤
35	Li Nan et al. 2017	8	OR=3.90，95%CI(2.92，5.20)	low	①④
30	Ruling Lu et al. 2018	11	OR=4.22，95%CI(2.86，6.23)	very low	①④⑤
28	Xu, Li-Fang et al. 2018	18	OR=2.83，95%CI(2.06，3.88)	very low	①④⑤
22	Xu Huayun et al. 2018	13	RR=1.19，95%CI(0.87，1.63)	low	①⑤
21	Huang, Wenfang et al. 2018	7	OR=2.63，95%CI(1.67，4.15)	very low	①④⑤
17	Jilin 2019	21	OR=3.38，95%CI(2.59，4.41)	Moderate	①
13	Xie Peng Peng et al 2019	14	RR=1.13，95%CI(1.02，1.24)	Moderate	①
12	Zhong Yizheng et al. 2019	10	RR=1.27，95%CI(1.13，1.44)	Moderate	①
11	Tung Yu Fong 2020	31	RR=1.26，95%CI(1.20，1.32)	Moderate	①
10	Li Nan et al. 2020	4	OR=3.42，95%CI(1.76，6.64)	very low	①④⑤
7	Limpibe 2020	18	RR=1.26，95%CI(1.17，1.36)	very low	①②④
4	Chan Kam Ming et al. 2020	6	RR=1.26，95%CI(1.15，1.37)	low	①④
2	Huang Ting et al. 2020	8	RR=1.25，95%CI(1.13，1.37)	very low	①④⑤

ovulation rate

	SR	include RCTs	ovulation rate	GRADE quality of evidence	Relegation factors
40	Yan, Lun et al. 2015	9	OR=2.18，95%CI(1.63，2.92)	very low	①④⑤
39	Xiao Chao 2016	8	RR=1.10，95%CI(0.87，1.39)	very low	①②④⑤
35	Li Nan et al. 2017	6	OR=2.70，95%CI(1.32，5.45)	very low	①②④⑤
21	Huang, Wenfang et al. 2018	6	OR=2.18，95%CI(1.77，2.68)	low	①⑤
20	Liu Ying et al. 2019	8	RR=1.34，95%CI(1.23，1.46)	low	①⑤
19	Original Bochao 2019	6	RR=0.97，95%CI(0.86，1.09)	very low	①②④⑤
17	Jilin 2019	14	OR=1.92，95%CI(1.40，2.64)	low	①②
13	Xie Peng Xin et al 2019	10	RR=1.17，95%CI(1.02，1.34)	low	①④
12	Zhong Yizheng et al. 2019	5	RR=1.18，95%CI(1.03，1.37)	low	①④
10	Li Nan et al. 2020	4	OR=3.91，95%CI(1.95，7.84)	very low	①④⑤
8	Du Xiu et al. 2020	6	RR=1.17，95%CI(1.03，1.34)	very low	①②④
7	Limpibe 2020	6	RR=1.31，95%CI(1.16，1.48)	low	①④
4	Chan Kam Ming et al. 2020	3	RR=1.21，95%CI(1.07，1.37)	very low	①④⑤

T

	SR	include RCTs	T	GRADE quality of evidence	Relegation factors
39	Xiao Chao 2016	8	SMD=-0.81，95%CI(-1.46，-0.16)	very low	①②④⑤
19	Original Bochao 2019	5	MD=-1.51，95%CI(-1.64，-1.37)	very low	①④⑤
17	Jilin 2019	24	SMD=-0.64，95%CI(-0.97，-0.36)	very low	①②⑤
13	Xie Peng Peng et al 2019	13	WMD=-0.93，95%CI(-1.38，-0.28)	very low	①②④⑤
12	Zhong Yizheng et al. 2019	9	SMD=-1.59，95%CI(-1.76，-1.41)	very low	①②④
11	Tung Yu Fong 2020	37	SMD=-0.40，95%CI(-0.65，-0.15)	very low	①②④
10	Li Nan et al. 2020	3	SMD=-0.68，95%CI(-3.99，2.62)	very low	①②④⑤
8	Du Xiu et al. 2020	5	RR=-0.53，95%CI(-0.90，-0.16)	very low	①②④
7	Limpibe 2020	19	SMD=-0.20，95%CI(-0.55，0.16)	very low	①②④⑤
4	Chan Kam Ming et al. 2020	2	MD=0.95，95%CI(0.15，1.75)	very low	①②④⑤

LH

	SR	include RCTs	LH	GRADE quality of evidence	Relegation factors
39	Xiao Chao 2016	7	SMD=-1.16，95%CI(-1.66，-0.66)	very low	①②④⑤
30	Ruling Lu et al. 2018	18	MD=-1.84，95%CI(-1.98，-1.70)	very low	①②⑤
19	Original Bochao 2019	5	MD=-6.72，95%CI(-7.32，-6.13)	very low	①④⑤
17	Jilin 2019	23	SMD=-0.55，95%CI(-0.74，-0.37)	low	①④
13	Xie Peng Peng et al 2019	13	WMD=-0.95，95%CI(-1.41，-0.52)	very low	①②④
12	Zhong Yizheng et al. 2019	9	SMD=-1.24，95%CI(-1.39，-1.08)	very low	①②④
11	Tung Yu Fong 2020	39	SMD=-0.38，95%CI(-0.59，-0.16)	very low	①②④
10	Li Nan et al. 2020	5	SMD=1.67，95%CI(-1.97，-1.37)	very low	①②④
7	Limpibe 2020	17	SMD=-0.78，95%CI(-1.22，-0.34)	very low	①②④
4	Chan Kam Ming et al. 2020	2	MD=7.55，95%CI(2.05，13.04)	very low	①②④⑤

FSH

17	Jilin 2019	19	SMD=0.12，95%CI(-0.29，-0.53)	very low	①②④
13	Xie Peng Peng et al 2019	11	WMD=-0.59，95%CI(-0.98，-0.20)	very low	①②④
12	Zhong Yizheng et al. 2019	8	SMD=0.66，95%CI(0.51，0.82)	low	①④
11	Tung Yu Fong 2020	37	SMD=0.01，95%CI(-0.22，0.25)	very low	①②④⑤
10	Li Nan et al. 2020	5	SMD=-1.67，95%CI(-3.05，-0.30)	very low	①②④⑤
4	Chan Kam Ming et al. 2020	2	MD=0.13，95%CI(-0.39，0.66)	very low	①②④⑤

adverse effects

	SR	include RCTs	adverse effects	GRADE quality of evidence	Relegation factors
40	Yan, Lun et al. 2015	4	OR=0.19，95%CI(0.08，0.46)	low	①⑤
39	Xiao Chao 2016	4	RD=-0.05，95%CI(-0.13，0.03)	very low	①②⑤
35	Li Nan et al. 2017	3	OR=0.07，95%CI(0.02，0.23)	very low	①②④⑤
28	Xu, Li-Fang et al. 2018	10	OR=0.26，95%CI(0.09，0.80)	low	①⑤
17	Jilin 2019	13	OR=0.26，95%CI(0.12，0.55)	very low	①②⑤
11	Tung Yu Fong 2020	8	RR=0.12，95%CI(0.06，0.25)	low	①④
7	Limpibe 2020	13	RR=0.36，95%CI(0.20，0.63)	low	①④

LH/FSH

	SR	include RCTs	L/F	GRADE quality of evidence	Relegation factors
39	Xiao Chao 2016	4	MD=-0.81，95%CI(-1.17，-0.45)	very low	①②④⑤
30	Ruling Lu et al. 2018	12	MD=-0.25，95%CI(-0.44，-0.06)	very low	①②⑤
17	Jilin 2019	11	SMD=-0.45，95%CI(-0.68，-0.23)	low	①②
13	Xie Peng Peng et al 2019	3	WMD=-1.04，95%CI(-1.78，-0.33)	very low	①②④⑤
11	Tung Yu Fong 2020	22	SMD=-0.39，95%CI(-0.60，-0.19)	very low	①②④
7	Limpibe 2020	14	MD=-0.37，95%CI(-0.53，-0.21)	very low	①②④
